# *XPD* gene polymorphism and host characteristics in the association with cutaneous malignant melanoma risk

**DOI:** 10.1038/sj.bjc.6601385

**Published:** 2004-01-20

**Authors:** A Baccarelli, D Calista, P Minghetti, B Marinelli, B Albetti, T Tseng, M Hedayati, L Grossman, G Landi, J P Struewing, M T Landi

**Affiliations:** 1 Genetic Epidemiology Branch, Division of Cancer Epidemiology and Genetics, NCI, NIH, DHHS, 6120 Executive Blvd., Bethesda, MD 20892-7236, USA; 2 Dermatology Unit, Bufalini Hospital, Viale Ghirotti 286, 47023 Cesena, Italy; 3 EPOCA, Epidemiology Research Centre, University of Milan, Via S. Barnaba, 8, Milan 20122, Italy; 4 Laboratory of Population Genetics, Centre for Cancer Research, NCI, NIH, DHHS, 41 Library Drive, Bethesda, MD 20892-5060, USA; 5 Department of Biochemistry, Bloomberg School of Public Health, The Johns Hopkins University, 615 North Wolfe Street, Baltimore, MD 21205, USA

**Keywords:** cutaneous malignant melanoma, *XPD*, *ERCC2*, DNA repair, age

## Abstract

We recently reported an association between low DNA repair capacity, measured through the host-cell reactivation assay, and melanoma risk in subjects with dysplastic naevi or low tanning ability. We investigated the genetic basis for these findings by analysing the Asp312Asn and Lys751Gln polymorphisms of the *XPD* (*ERCC2*) DNA repair gene in the same subjects. Similar to our previous report, no significant association between *XPD* polymorphisms and melanoma risk was found in 176 melanoma cases and 177 controls (odds ratio (OR)=1.5, 95% confidence interval (CI)=0.9–2.5 for 312Asn; OR=1.3, 95% CI=0.8–2.1 for 751Gln, adjusted for age, gender, dysplastic naevi and pigmentation characteristics). However, *XPD* variants were associated with increased risk in older (>50 years) subjects (OR=3.4, 95% CI=1.6–7.3 for 312Asn; OR=2.3, 95% CI=1.1–4.9 for 751Gln). The 751Gln allele was associated with elevated melanoma risk among subjects without dysplastic naevi (OR=2.6, 95% CI=1.1–6.4). Subjects with low tanning ability and *XPD* variants exhibited a nonsignificant increase of melanoma risk (OR=2.3, 95% CI=0.7–7.0 for 312Asn; OR=3.0, 95% CI=1.0–8.8 for 751Gln). DNA repair capacity was slightly decreased in subjects carrying 751Gln alleles. *XPD* variants may modify melanoma risk in subjects with specific host characteristics, such as older age, lack of dysplastic naevi or low tanning ability.

Between 1940 and 2000, descriptive studies documented a progressive worldwide rise in the incidence of cutaneous malignant melanoma. Melanoma risk increases with age (Kosary *et al*, 1996; Gilchrest *et al*, 1999) and other risk factors, such as sunlight exposure (IARC, 1992), particularly intermittent (Gilchrest *et al*, 1999), family history of melanoma, dysplastic naevi or atypical naevi, number of naevi, skin sensitivity to sun, freckling, fair hair, eye and skin colour (Tucker *et al*, 1997; Landi *et al*, 2001). Individuals with inherited defects in nucleotide excision repair (eg, patients with xeroderma pigmentosum, XP) have low repair of UV-induced DNA lesions and are at extremely high risk of skin cancers, including melanoma (Berneburg and Lehmann, 2001). In healthy subjects, polymorphisms in DNA repair genes may be associated with altered DNA repair capacity and susceptibility to cancer (Benhamou and Sarasin, 2002).

The XP complementation group D (XPD, also known as ERCC2) protein, a subunit of the Transcription Factor IIH (TFIIH), is involved in DNA unwinding during DNA repair and initiation of basal transcription (Berneburg and Lehmann, 2001). Several single nucleotide polymorphisms (SNPs) in *XPD* gene exons have been identified (Benhamou and Sarasin, 2002). The nonsilent variants at codons 312 (exon10, G>A, Asp312Asn) and 751 (Exon23, A>C, Lys751Gln) are amenable to investigation in epidemiological studies in Caucasians, given their high frequency (Benhamou and Sarasin, 2002). The *XPD* 751Gln variant substantially modifies the amino-acid electronic configuration in a domain important for the interaction with the helicase activator p44 and may produce the most relevant change in XPD function (Coin *et al*, 1998; Benhamou and Sarasin, 2002). Aging has been associated with depressed *in situ* repair of UV-specific DNA lesions in the skin of subjects carrying the *XPD* variants 312Asn and 751Gln (Hemminki *et al*, 2001), who may therefore be at higher risk for cancer at an older age (Dybdahl *et al*, 1999; Sturgis *et al*, 2000). We recently showed that specific host characteristics, such as presence of dysplastic naevi and low tanning ability, modify the risk of melanoma associated with low DNA repair capacity (Landi *et al*, 2002). We report here on (i) the association between the Asp312Asn and Lys751Gln *XPD* polymorphisms and cutaneous malignant melanoma risk, (ii) the effect modification of age, presence of dysplastic naevi and tanning ability, and (iii) the association between *XPD* polymorphisms and DNA repair capacity, in the same subjects.

## MATERIALS AND METHODS

### Study subjects

From December 1994 to January 1999, we recruited 183 (87 male and 96 female) cases with incident nonfamilial cutaneous malignant melanoma (of any stage) and 179 (89 male and 90 female) controls between the ages of 17 and 77 years at the Dermatology Unit of the Bufalini Hospital in Cesena, Italy, as previously described (Landi *et al*, 2001,^2002^). The Hospital examined approximately 85% of all the melanoma patients of the Northern Marche and Southern Romagna areas, from which all study subjects came from (Landi *et al*, 2002). Approximately 95% of the cases and 83% of the controls agreed to participate in the study. Control subjects, frequency-matched to the cases by decade of age and gender, were recruited among spouses or close friends of the cases (*n*=134), outpatients with minor accidental trauma (*n*=14) and healthy volunteers from the hospital personnel (*n*=34). The exclusion of the trauma patients and/or the hospital personnel volunteers from the analysis did not change the results. We obtained the study approval from the Bufalini Hospital's Ethical Committee and informed written consent from all participants. Trained interviewers administered a standardised in-person questionnaire and a single dermatologist examined all study subjects. The tanning ability was ascertained through the following question: ‘After repeated and prolonged exposure to sunlight, your skin (1) becomes very tanned, (2) becomes medium tanned, (3) becomes hardly tanned, (4) has a tendency to peel, or (5) has absolutely no change'.

Because no one selected choice 4 and only three selected choice 5, the answers were categorised into three groups: high tanning response (choice 1), medium tanning response (choice 2), and low tanning response (choices 3 and 5) (Landi *et al*, 2002).

An expert oncologist, blinded to melanoma status, assessed dysplastic naevus diagnoses and naevus counts in standardised photographs of the subjects' backs, as previously described (Landi *et al*, 2002). In total, 15 cases and 15 controls had either no photographs or uncertain dysplastic naevus diagnosis. A three-category variable (dysplastic naevi, no dysplastic naevi, unknown/uncertain dysplastic naevi) was used in the statistical models. Naevi and dysplastic naevi tend to disappear with age, making their diagnosis difficult to assess in older individuals (Tucker *et al*, 1997). We, therefore, classified all subjects (47 cases and 28 controls) older than 60 years of age as with uncertain dysplastic naevus status (Landi *et al*, 2002). However, the use in the analysis of dysplastic naevus data from these older subjects did not change the results.

### DNA extraction and *XPD* genotyping

Genomic DNA was extracted from 1–2 × 10^6^ cryopreserved lymphocytes. We used the Nucleon Extraction and Purification kit (Amersham LIFE SCIENCE, UK), following the manufacturer's instructions. Using lymphocyte DNA as template, we genotyped the Asp312Asn SNP using a Taqman 5′-nuclease assay and the Lys751Gln with a PCR–RFLP assay. Asp312Asn was genotyped in a 25-*μ*l reaction containing Taqman Universal Master Mix (Applied Biosystems, Foster City, CA, USA) run on an ABI 7700 Sequence-Detection system (Applied Biosystems, Foster City, CA, USA). The reaction included the primers 5′-CTCCgCAggATCAAAgAgACA-3′ and 5′-TCTgCgAggAgACgCTATCA-3′ at 900 nM, and the probes 5′-VIC-CCgTgCTgCCCgACgAAgT-TAMRA-3′ recognising the Asp(G) allele and 5′-6FAM-CCgTgCTgCCCAACgAAgTg-TAMRA-3′ recognising the Asn(A) allele, at 200 nM and 10 ng of genomic DNA as template. No template controls and samples verified to have all three possible genotypes were included with each reaction plate. Genotypes for Asp312Asn were successfully determined in 164 cases and 172 controls. The Lys751Gln polymorphic site was amplified using the primers 5′-ATCCTgTCCCTACTggCCATTC-3′ and 5′-TgTggACgTgACAgTgAgAAAT-3′ in 50 *μ*l polymerase chain reaction (PCR) reactions containing: 10 mM Tris-HCl (pH 8.8 at 25°C), 1.5 mM MgCl_2_, 50 mM KCl, 0.1% Triton X-100, 0.2 mM each deoxynucleotide triphosphate, 20 pmoles of each primer, 2 U of *Taq* DNA Polymerase Dynazyme (Finzymes, ESPOO, Finland) and 50–100 ng of genomic DNA. The cycling conditions were: initial denaturation at 95°C for 3 min, 30 cycles of denaturation at 94°C for 45 s, primer annealing at 60°C for 45 s, primer extension at 72°C for 1 min and final extension at 72°C for 5 min. The PCR product was digested with 10–15 U of *PstI* enzyme (Promega, Madison, WI, USA) in a 25-*μ*l-reaction mixture for 2 h at 37°C, as suggested by the manufacturer, and separated on a 2% agarose gel. In addition to a *PstI* site away from the polymorphism that serves as internal control for digestion, the Lys(A) allele has a second *PstI* restriction site. The three possible genotypes are defined by three distinct banding patterns: AA (100- and 224-bp fragments), AC (66-, 100-, 158- and 224-bp fragments) and CC (66-, 100- and 158-bp fragments). Genotypes for Lys751Gln were successfully determined in 176 cases and 177 controls. For quality control purposes, DNA samples from 335 subjects who had been tested for the Lys751Gln polymorphisms were genotyped by an independent laboratory. Between-laboratory agreement was equal to 97.3%.

### Host-cell reactivation (HCR) assay

We measured DNA repair capacity in cryopreserved lymphocytes by the CAT-gene based HCR assay, as previously described (Athas *et al*, 1991; Landi *et al*, 2002). CAT activity>1000 c.p.m. in unirradiated cells is required to measure accurately the DNA repair capacity with a signal-to-noise ratio at least two-fold higher than the background level (200 c.p.m.) in cells with irradiated plasmids (Landi *et al*, 2002). DNA repair capacity measured in lymphocytes with baseline CAT activity<1000 c.p.m. (44 cases and 32 controls) was not considered in the analysis.

All laboratory analyses were performed blinded to the case status.

### Statistical analysis

We calculated adjusted odds ratios (ORs) and 95% confidence intervals (CIs) by unconditional multiple logistic regression models that included the matching variables (i.e., age and gender), presence of dysplastic naevi, skin colour, tanning ability and eye colour as independent variables. Because of the relatively small number of subjects with the homozygous variant genotypes, we used two-category variables (consensus *vs* heterozygous/homozygous) in logistic regression analysis. The models with three-category variables were not substantially different from the models with two-category variables (*P⩾*0.17, likelihood ratio tests). We used the likelihood ratio test to test for interactions, which were also evaluated using a case-only analysis (Yang *et al*, 1997). The case-only approach confirmed the results of the case–control analysis. Here, we report the analysis based on cases and controls. Age was a matching variable and, therefore, we could not estimate its main effect on melanoma risk in the logistic models including the interaction between age and the *XPD* polymorphisms. Hardy–Weinberg equilibrium was tested using the asymptotic Pearson's *χ*^2^-test. We used the Fisher's exact test to compare proportions and the Spearman's rank correlation statistics to assess linear correlation between variables. All statistical tests were two-sided. The Stata 7.0 statistical package was used for all analyses (Stata Corporation, College Station, TX, USA).

## RESULTS

### Distribution of the *XPD* polymorphisms

Frequencies of *XPD* 312Asn and 751Gln alleles were 0.396 and 0.403, respectively, in cases, and 0.398 and 0.427, respectively, in controls. The distribution of both polymorphisms among controls was consistent with the Hardy–Weinberg equilibrium (*P*=0.30 for Asp312Asn and *P*=0.81 for Lys751Gln), also when the subjects were subdivided in age, dysplastic naevus status or tanning ability categories. Consistently with previous reports (Butkiewicz *et al*, 2001; Zhou *et al*, 2002), carriers of the 312Asp/Asp genotype tended to have the 751Lys/Lys genotype (test for the association between Asp312Asn and Lys751Gln genotypes: *P*=2.1 × 10^−24^ in controls, and *P*=1.1 × 10^−22^ in cases).

In controls, *XPD* genotypes were not associated with age, gender or the strongest risk factors for melanoma in this population (Landi *et al*, 2001), that is, eye colour, skin colour, tanning ability to prolonged and repeated sun exposure, and presence of dysplastic naevi (Table 1

**Table 1:** Characteristics of cases and controls by *XPD* Asp312Asn and Lys751Gln polymorphisms

	***XPD* 312**						***XPD* 751**					
	**Case**			**Control**			**Case**			**Control**		
	**Asp/Asp (G/G)**	**Asp/Asn (G/A)**	**Asn/Asn (A/A)**	**Asp/Asp (G/G)**	**Asp/Asn (G/A)**	**Asn/Asn (A/A)**	**Lys/Lys (A/A)**	**Lys/Gln (A/C)**	**Gln/Gln (C/C)**	**Lys/Lys (A/A)**	**Lys/Gln (A/C)**	**Gln/Gln (C/C)**
	***n*=52**	***n*=94**	***n*=18**	***n*=59**	***n*=89**	***n*=24**	***n*=58**	***n*=94**	***n*=24**	***n*=59**	***n*=85**	***n*=33**
*Age (years)*												
17–49	31	43	6	28	58	16	34	42	9	30	54	21
50–77	21	51	12	31	31	8	24	52	15	29	31	12
												
*Gender*												
Male	28	41	9	30	43	12	34	37	13	33	40	16
Female	24	53	9	29	46	12	24	57	11	26	45	17
												
*Eye colour*^a^												
Dark	13	27	2	19	38	11	19	22	4	20	36	17
Medium	24	54	12	33	42	10	27	55	16	32	40	13
Light	15	13	4	7	8	3	12	17	4	7	8	3
												
*Skin colour*												
Dark/medium	22	43	6	37	62	16	26	41	9	41	56	23
Light	29	51	12	22	27	8	31	53	15	18	29	10
												
*Tanning ability to prolonged sun exposure*												
High/medium	36	57	10	51	71	21	41	54	16	47	70	29
Low	15	33	8	8	15	2	16	37	7	11	11	4
												
*Dysplastic naevi*^b^												
No	12	32	8	29	60	14	9	34	12	33	52	20
Yes	23	27	5	11	11	6	26	29	4	10	14	5

a Dark – black or dark brown. Medium – light brown, brown-green, green or blue-green. Light – light blue, dark blue or grey.

b Subjects older than 60 years of age were considered as with uncertain dysplastic naevus status. The total number of subjects may vary across variables due to missing values.

). In addition, *XPD* genotypes were not associated with hair colour, freckling, skin response to 30 min of sun exposure, naevus number, lifetime number of severe sunburns, and lifetime or childhood cumulative hours of sun exposure during vacation (data not shown).

### *XPD* polymorphisms and melanoma risk

Overall, the *XPD* polymorphisms were not significantly associated with melanoma risk (Table 2

**Table 2:** *XPD* Asp312Asn and Lys751Gln polymorphisms and risk of cutaneous malignant melanoma in the overall analysis and by age, presence of dysplastic naevi or tanning ability

	***XPD* 312**					***XPD* 751**				
	**Genotype**	**Case**	**Control**	**OR^a^**	**(95% CI)^a^**	**Genotype**	**Case**	**Control**	**OR^a^**	**(95% CI)^a^**
*Overall analysis*										
	Asp/Asp (G/G)	52	59	1.0	Ref.	Lys/Lys (A/A)	58	59	1.0	Ref.
	Asp/Asn (G/A) Asn/Asn (A/A)	112	113	1.5	(0.9-2.5)	Lys/Gln (A/C) Gln/Gln (C/C)	118	118	1.3	(0.8–2.1)
										
*By age (years)*										
17–49	Asp/Asp (G/G)	31	28	1.0	Ref.	Lys/Lys (A/A)	34	30	1.0	Ref.
17–49	Asp/Asn (G/A) Asn/Asn (A/A)	49	74	0.7	(0.4-1.5)	Lys/Gln (A/C) Gln/Gln (C/C)	51	75	0.7	(0.4–1.5)
50–77	Asp/Asp (G/G)	21	31	1.0	Ref.	Lys/Lys (A/A)	24	29	1.0	Ref.
50–77	Asp/Asn (G/A) Asn/Asn (A/A)	63	39	3.4	(1.6–7.3)	Lys/Gln (A/C) Gln/Gln (C/C)	67	43	2.3	(1.1–4.9)
Test of heterogeneity of ORs^b^					*P*=0.004					*P*=0.03
										
*By presence of dysplastic navi*^c^										
No	Asp/Asp (G/G)	12	29	1.0	Ref.	Lys/Lys (A/A)	9	33	1.0	Ref.
No	Asp/Asn (G/A) Asn/Asn (A/A)	40	74	1.4	(0.6-3.2)	Lys/Gln (A/C) Gln/Gln (C/C)	46	72	2.6	(1.1–6.4)
Yes	Asp/Asp (G/G)	23	11	1.0	Ref.	Lys/Lys (A/A)	26	10	1.0	Ref.
Yes	Asp/Asn (G/A) Asn/Asn (A/A)	32	17	0.9	(0.3–2.4)	Lys/Gln (A/C) Gln/Gln (C/C)	33	19	0.6	(0.2–1.6)
Test of heterogeneity of ORs^b^					*P*=0.52					*P*=0.03
										
*By tanning ability*										
High/medium	Asp/Asp (G/G)	36	51	1.0	Ref.	Lys/Lys (A/A)	41	47	1.0	Ref.
High/medium	Asp/Asn (G/A) Asn/Asn (A/A)	67	92	1.3	(0.7–2.4)	Lys/Gln (A/C) Gln/Gln (C/C)	70	99	1.0	(0.6–1.8)
Low	Asp/Asp (G/G)	15	8	1.0	Ref.	Lys/Lys (A/A)	16	11	1.0	Ref.
Low	Asp/Asn (G/A) Asn/Asn (A/A)	41	17	2.3	(0.7-7.0)	Lys/Gln (A/C) Gln/Gln (C/C)	44	15	3.0	(1.0-8.8)
Test of heterogeneity of ORs^b^					*P*=0.39					*P*=0.08

a Odds ratios (ORs) and 95% confidence intervals (95% CIs) adjusted for age, gender, dysplastic naevus status, skin colour, tanning ability and eye colour.

b Likelihood-ratio test for interaction with XPD polymorphism.

c Subjects older than 60 years of age were considered as with uncertain dysplastic naevus status

). Subjects with at least one 312Asn variant allele had an OR for melanoma of 1.5 (95% CI=0.9–2.5, adjusted for age, gender, presence of dysplastic naevi, skin colour, tanning ability, and eye colour) compared with individuals with the 312Asp/Asp genotype. Subjects with at least one 751Gln variant allele had an OR of 1.3 (95% CI=0.8–2.1) in comparison with subjects carrying the 751Lys/Lys genotype.

Upon dividing the subjects into two age groups, we found an increased melanoma risk for older subjects carrying variant alleles (Table 2). Among subjects with age⩾50 years, those with at least one 312Asn allele had an OR of 3.4 (95% CI=1.6–7.3). Subjects with the 751Gln variant had OR=2.3 (95% CI=1.1–4.9). In subjects <50 years of age, the XPD variant alleles were not associated with melanoma (OR=0.7, 95% CI=0.4–1.5 for 312Asn; OR=0.7, 95% CI=0.4–1.5 for 751Gln). The relative odds due to the *XPD* variants were significantly higher in older subjects (*P*=0.004 for 312Asn; *P*=0.03 for 751Gln). We used the 50-years cutoff to define the age categories in order to compare our results with those reported in previous studies (Sturgis *et al*, 2000; Hemminki *et al*, 2001). We found similar results using the actual median age of all the subjects (48 years) or the median age of the controls (45 years) (data not shown).

After the subjects were stratified according to the presence of dysplastic naevi (Table 2), individuals without dysplastic naevi and with the 751Gln/Lys or 751Gln/Gln genotypes showed an increased melanoma risk (OR=2.6, 95% CI=1.1–6.4) compared to those without dysplastic naevi and with the 751Lys/Lys genotype. Among subjects without dysplastic naevi, the association between the *XPD* polymorphism and melanoma risk appeared limited to older subjects. When we repeated the analysis in subjects who were 50 years of age or older, having variant alleles at codon 751 was associated with the highest risk of melanoma among individuals without dysplastic naevi (OR=13.9, 95% CI=1.4–136.3). Among subjects with dysplastic naevi (Table 2), those with at least one 751Gln variant allele did not show a different risk (OR=0.6, 95% CI=0.2–1.6) from those with the 751Lys/Lys genotype. The effect of the 751Gln variant was significantly higher among subjects without dysplastic naevi in comparison with subjects with dysplastic naevi (*P*=0.03). In fact, subjects with dysplastic naevi had higher relative odds for melanoma, but they did not significantly vary by *XPD* status: when compared with subjects without dysplastic naevi and with the 751Lys/Lys genotype, individuals with dysplastic naevi and the 751Lys/Lys genotype had OR=10.9 (95% CI=3.6–33.7); those with dysplastic naevi and either the 751Gln/Lys or 751Gln/Gln genotype had OR=6.7 (95% CI=2.4–18.2). Among older subjects with dysplastic naevi, the variant alleles had lower relative odds (OR=2.3, 95%CI=0.3–18.4 for subjects with dysplastic naevi and 751Gln/Lys or 751Gln/Gln genotype *vs* subjects with dysplastic naevi and 751Lys/Lys genotype) than in subjects without dysplastic naevi. Odds ratios for melanoma were not associated with the Lys751Gln polymorphism among subjects younger than 50 years of age, independently of their dysplastic naevus status.

Melanoma risk associated with the *XPD* 312Asn variant was not increased (Table 2) and did not significantly vary by dysplastic naevus status (*P*=0.52).

Among subjects with low tanning ability, the *XPD* variant alleles (Table 2) exhibited an increased risk for melanoma (OR=2.3, 95% CI=0.7–7.0 for 312Asn; OR=3.0, 95% CI=1.0–8.8 for 751Gln). In subjects with medium or high tanning ability, we observed no association of melanoma risk with the 312Asn (OR=1.3, 95% CI=0.7–2.4) or 751Gln (OR=1.0, 95% CI=0.6–1.8) variant. However, the risk modification by tanning ability was not statistically significant (*P*⩾0.08). Even though based on small number of subjects, we found similar results after dividing the subjects with low tanning ability in two groups with high or low lifetime recreational sun exposure (data not shown).

### *XPD* polymorphisms and DNA repair capacity

We evaluated whether global DNA repair capacity, measured through the HCR assay, varied in subjects with different *XPD* genotypes. DNA repair capacity exhibited a nonsignificant trend in the association with the Lys751Gln polymorphism. The median DNA repair capacity after 350 J m^−2^ UV-irradiation was 19.5% in subjects (*n*=88) with the 751Lys/Lys genotype, 18.5% in subjects (*n*=144) with the 751Gln/Lys genotype, and 17.3% in subjects (*n*=45) with the 751Gln/Gln genotype (*P*=0.55). No association was present between Asp312Asn and DNA repair capacity. We adjusted for cumulative hours of sun exposure during vacation, repeated the analysis in cases, controls, and/or subgroups with different age, dysplastic naevus status or tanning ability, and did not find any significant correlation between Asp312Asn or Lys751Gln and DNA repair capacity.

## DISCUSSION

Previous investigations in noncancer subjects with different ethnic backgrounds reported variable frequencies of the 312Asn and 751Gln alleles (Goode *et al*, 2002). We found a high frequency of the two *XPD* variants in this Italian population, as previously described by other investigators (Matullo *et al*, 2001) for the 751Gln polymorphism.

Similar to our previous report on DNA repair capacity and melanoma (Landi *et al*, 2002), we did not find a significant overall association between melanoma risk and the *XPD* polymorphisms. However, both 312Asn and 751Gln alleles were associated with increased melanoma risk in subjects who were 50 years of age or older. Recently, Hemminki *et al* (2001) showed that the Asp312Asn and Lys751Gln polymorphisms might interact with age in their association with repair rates of UV-induced lesions in the skin. In the Hemminki's study, subjects ⩾50 years of age carrying the *XPD* variants exhibited lower DNA repair.

To date, only two studies have investigated the association between melanoma risk and *XPD* polymorphisms. A small study that compared the results from 28 Stage I melanoma patients with 33 samples from healthy blood donors found an overrepresentation of the 751Lys allele among the melanoma cases (Tomescu *et al*, 2001). This first investigation that did not analyse the Asp312Asn polymorphism was restricted to subjects younger than 50 years of age. In our study, the proportion of the 751Lys/Lys genotype was moderately higher among cases than controls in younger subjects (Table 2). A second investigation conducted in 125 melanoma cases and 211 cadaveric renal transplant donors failed to find an association between Asp312Asn or Lys751Gln and melanoma (Winsey *et al*, 2000). The median age of the cases was 52 years; the age of the control group was not reported. No data on the interaction between *XPD* variants and age or other risk factors were provided in these two studies. Similar to our results, two additional investigations on basal cell carcinoma (Dybdahl *et al*, 1999) and head and neck cancer (Sturgis *et al*, 2000) suggested that carriers of the *XPD* 751Gln allele may be at risk for cancer at an older age. None of the two studies evaluated the Asp312Asn polymorphism.

Gilchrest *et al* (1999) suggested that age plays a major role in vulnerability to photocarcinogens. Melanoma risk exhibits a steep increase with age. In the study area, melanoma incidence rates, which we calculated from available data (Parkin *et al*, 1997), were equal to 6.1/100 000 person-years in subjects between 15 and 49 years of age and 17.5/100 000 person-years between 50 and 74 years. Cumulative DNA damage during aging is the result of errors in DNA replication occurring at low but finite rate or of incompletely repaired DNA damage (Yaar and Gilchrest, 2001). As age increases, subjects accumulate more opportunities for DNA damage due to prolonged exposure to UV radiation and other carcinogens. Furthermore, defences against sunlight exposure, such as skin pigmentation (Yaar and Gilchrest, 2001), epidermal thickness (Yaar and Gilchrest, 2001), and DNA repair efficiency (Wei *et al*, 1993; Yaar and Gilchrest, 1998) may decrease with age. In older subjects, the equilibrium between DNA damage and repair may be altered and further destabilised in presence of variant alleles in DNA repair genes, which may be otherwise negligible. The genetic alterations and the possible sequence of mutations needed for the progression to melanoma are still to be defined. Bennet (2003) recently proposed a speculative model for the genetic basis of primary melanoma in relation to cell senescence. In this model, early gene mutations leading to melanocyte proliferation, such as those in the *RAS*, protein tyrosine kinase or *BRAF* genes, are followed by lesions in the *p16/RB1* pathway. These alterations may be associated with the development of common or dysplastic naevi and, eventually, with the radial growth phase of malignant melanoma. Additional molecular events possibly linked to apoptosis suppression, such as *PTEN* loss, overexpression of a number of protein tyrosine kinases and *RAS* or beta-catenin activation, could be required for the progression to invasive melanoma. Further research is needed to assess how variations in XPD and, in general, DNA repair function affect the molecular events involved in melanoma development.

In a previous study on the same population, we showed an interaction in the association with melanoma risk between the overall DNA repair capacity, measured using the HCR assay, and presence of dysplastic naevi or low tanning ability (Landi *et al*, 2002). In the present study, the effect of the *XPD* 751Gln allele was evident in subjects without dysplastic naevi, particularly at older age. Presence of dysplastic naevi did not appear to modify the association between *XPD* Lys751Gln polymorphism and melanoma risk in subjects younger than 50 years of age. The presence of dysplastic naevi is one of the strongest risk factors for melanoma. The association between dysplastic naevi and melanoma in our population was also very strong (Landi *et al*, 2001) and this could have overwhelmed the effect of *XPD* variants in older subjects with dysplastic naevi. Caution is needed in the appraisal of these results because the relatively small sample size limited the assessment of the association between melanoma and *XPD* genotype when subjects were categorised at the same time by dysplastic naevus status and age. Naevi and dysplastic naevi tend to disappear with age, making their diagnosis difficult to assess in older individuals (Tucker *et al*, 1997). To be conservative, we classified all subjects older than 60 years of age as of uncertain dysplastic naevus status. However, the inclusion in the statistical analysis of the dysplastic naevus diagnoses in older subjects did not change the results.

As for tanning ability, similar to what we found in the association between DNA repair capacity and melanoma (Landi *et al*, 2002), there was a nonsignificantly increased risk of melanoma in subjects with both low tanning ability and a variant genotype. Subjects with a low tanning ability may be at increased risk of DNA damage, because they have lower protection against UV-exposure. A less efficient DNA repair may increase melanoma risk in these subjects.

We found a nonsignificant association between the overall DNA repair capacity and Lys751Gln polymorphism. This finding is consistent with previous investigations that measured DNA repair capacity by the HCR assay on peripheral lymphocytes of healthy subjects (Seker *et al*, 2001; Spitz *et al*, 2001) or on lymphoblastoid cell lines (Qiao *et al*, 2002b). The sensitivity of the assay measuring DNA repair capacity may have been a limiting factor in the assessment of the association. A recent study using the HCR assay based on the luciferase reporter gene found, as we did, significantly lower levels of DNA repair capacity in 751Gln/Gln than in 751Lys/Lys subjects, but in this case the association was statistically significant (Qiao *et al*, 2002a).

In conclusion, the *XPD* 312Asn and 751Gln variant alleles were associated with an increased risk for melanoma in subjects older than 50 years of age. The presence of dysplastic naevi and low tanning ability may also affect the association between *XPD* polymorphisms and melanoma risk. As with all statistical interactions that are identified for the first time, replication in an independent study is necessary. Future studies, assessing multiple genes involved in nucleotide excision repair, may help evaluate the genetic basis of the interplay between DNA repair and age or other host characteristics.

## References

[bib1] Athas WF, Hedayati MA, Matanoski GM, Farmer ER, Grossman L (1991) Development and field-test validation of an assay for DNA repair in circulating human lymphocytes. Cancer Res 51: 5786–5793 1933849

[bib2] Benhamou S, Sarasin A (2002) ERCC2/XPD gene polymorphisms and cancer risk. Mutagenesis 17: 463–469 12435843 10.1093/mutage/17.6.463

[bib3] Bennet DC (2003) Human melanocyte senescence and melanoma susceptibility genes. Oncogene 19: 3063–3069 10.1038/sj.onc.1206446 12789281

[bib4] Berneburg M, Lehmann AR (2001) Xeroderma pigmentosum and related disorders: defects in DNA repair and transcription. Adv Genet 43: 71–102 11037299 10.1016/s0065-2660(01)43004-5

[bib5] Butkiewicz D, Rusin M, Enewold L, Shields PG, Chorazy M, Harris CC (2001) Genetic polymorphisms in DNA repair genes and risk of lung cancer. Carcinogenesis 22: 593–597 11285194 10.1093/carcin/22.4.593

[bib6] Coin F, Marinoni JC, Rodolfo C, Fribourg S, Pedrini AM, Egly JM (1998) Mutations in the XPD helicase gene result in XP and TTD phenotypes, preventing interaction between XPD and the p44 subunit of TFIIH. Nat Genet 20: 184–188 9771713 10.1038/2491

[bib7] Dybdahl M, Vogel U, Frentz G, Wallin H, Nexo BA (1999) Polymorphisms in the DNA repair gene XPD: correlations with risk and age at onset of basal cell carcinoma. Cancer Epidemiol Biomarkers Prev 8: 77–81 9950243

[bib8] Gilchrest BA, Eller MS, Geller AC, Yaar M (1999) The pathogenesis of melanoma induced by ultraviolet radiation. N Engl J Med 340: 1341–1348 10219070 10.1056/NEJM199904293401707

[bib9] Goode EL, Ulrich CM, Potter JD (2002) Polymorphisms in DNA repair genes and associations with cancer risk. Cancer Epidemiol Biomarkers Prev 11: 1513–1530 12496039

[bib10] Hemminki K, Xu G, Angelini S, Snellman E, Jansen CT, Lambert B, Hou SM (2001) XPD exon 10 and 23 polymorphisms and DNA repair in human skin *in situ*. Carcinogenesis 22: 1185–1188 11470747 10.1093/carcin/22.8.1185

[bib11] IARC (1992) Solar and Ultraviolet Radiation. Lyon: International Agency for Research on Cancer

[bib12] Kosary CL, Ries LAG, Miller BA, Hankey BF, Harras A, Edwards BK (1996) SEER Cancer Statistics Review, 1973–1992: Table and Graphs. Bethesda, MD: National Cancer Institute

[bib13] Landi MT, Baccarelli A, Calista D, Pesatori A, Fears T, Tucker MA, Landi G (2001) Combined risk factors for melanoma in a Mediterranean population. Br J Cancer 85: 1304–1310 11720465 10.1054/bjoc.2001.2029 PMC2375242

[bib14] Landi MT, Baccarelli A, Tarone RE, Pesatori A, Tucker MA, Hedayati M, Grossman L (2002) DNA repair, dysplastic nevi, and sunlight sensitivity in the development of cutaneous malignant melanoma. J Natl Cancer Inst 94: 94–101 11792747 10.1093/jnci/94.2.94

[bib15] Matullo G, Palli D, Peluso M, Guarrera S, Carturan S, Celentano E, Krogh V, Munnia A, Tumino R, Polidoro S, Piazza A, Vineis P (2001) XRCC1, XRCC3, XPD gene polymorphisms, smoking and (32)P-DNA adducts in a sample of healthy subjects. Carcinogenesis 22: 1437–1445 11532866 10.1093/carcin/22.9.1437

[bib16] Parkin DM, Whelan SL, Ferlay J, Raymond L, Young J (1997) Italy, Romagna. In Cancer Incidence in Five Continents, Vol VII pp 554–557. Lyon: International Agency for Research on Cancer

[bib17] Qiao Y, Spitz MR, Guo Z, Hadeyati M, Grossman L, Kraemer KH, Wei Q (2002a) Rapid assessment of repair of ultraviolet DNA damage with a modified host-cell reactivation assay using a luciferase reporter gene and correlation with polymorphisms of DNA repair genes in normal human lymphocytes. Mutat Res 509: 165–174 12427537 10.1016/s0027-5107(02)00219-1

[bib18] Qiao Y, Spitz MR, Shen H, Guo Z, Shete S, Hedayati M, Grossman L, Mohrenweiser H, Wei Q (2002b) Modulation of repair of ultraviolet damage in the host-cell reactivation assay by polymorphic XPC and XPD/ERCC2 genotypes. Carcinogenesis 23: 295–299 11872635 10.1093/carcin/23.2.295

[bib19] Seker H, Butkiewicz D, Bowman ED, Rusin M, Hedayati M, Grossman L, Harris CC (2001) Functional significance of XPD polymorphic variants: attenuated apoptosis in human lymphoblastoid cells with the XPD 312 Asp/Asp genotype. Cancer Res 61: 7430–7434 11606376

[bib20] Spitz MR, Wu X, Wang Y, Wang LE, Shete S, Amos CI, Guo Z, Lei L, Mohrenweiser H, Wei Q (2001) Modulation of nucleotide excision repair capacity by XPD polymorphisms in lung cancer patients. Cancer Res 61: 1354–1357 11245433

[bib21] Sturgis EM, Zheng R, Li L, Castillo EJ, Eicher SA, Chen M, Strom SS, Spitz MR, Wei Q (2000) XPD/ERCC2 polymorphisms and risk of head and neck cancer: a case-control analysis. Carcinogenesis 21: 2219–2223 11133811 10.1093/carcin/21.12.2219

[bib22] Tomescu D, Kavanagh G, Ha T, Campbell H, Melton DW (2001) Nucleotide excision repair gene XPD polymorphisms and genetic predisposition to melanoma. Carcinogenesis 22: 403–408 11238179 10.1093/carcin/22.3.403

[bib23] Tucker MA, Halpern A, Holly EA, Hartge P, Elder DE, Sagebiel RW, Guerry D, Clark WHJ (1997) Clinically recognized dysplastic nevi. A central risk factor for cutaneous melanoma. JAMA 277: 1439–1444 9145715

[bib24] Wei Q, Matanoski GM, Farmer ER, Hedayati MA, Grossman L (1993) DNA repair and aging in basal cell carcinoma: a molecular epidemiology study. Proc Natl Acad Sci USA 90: 1614–1618 8434025 10.1073/pnas.90.4.1614 PMC45925

[bib25] Winsey SL, Haldar NA, Marsh HP, Bunce M, Marshall SE, Harris AL, Wojnarowska F, Welsh KI (2000) A variant within the DNA repair gene XRCC3 is associated with the development of melanoma skin cancer. Cancer Res 60: 5612–5616 11059748

[bib26] Yaar M, Gilchrest BA (1998) Aging versus photoaging: postulated mechanisms and effectors. J Invest Dermatol Symp Proc 3: 47–51 9732058

[bib27] Yaar M, Gilchrest BA (2001) Ageing and photoageing of keratinocytes and melanocytes. Clin Exp Dermatol 26: 583–591 11696062 10.1046/j.1365-2230.2001.00895.x

[bib28] Yang Q, Khoury MJ, Flanders WD (1997) Sample size requirements in case-only designs to detect gene-environment interaction. Am J Epidemiol 146: 713–720 9366618 10.1093/oxfordjournals.aje.a009346

[bib29] Zhou W, Liu G, Miller DP, Thurston SW, Xu LL, Wain JC, Lynch TJ, Su L, Christiani DC (2002) Gene–environment interaction for the ERCC2 polymorphisms and cumulative cigarette smoking exposure in lung cancer. Cancer Res 62: 1377–1381 11888908

